# Research of CO_2_-Soluble Surfactants for Enhanced Oil Recovery: Review and Outlook

**DOI:** 10.3390/molecules28248042

**Published:** 2023-12-11

**Authors:** Shisheng Liang, Wenli Luo, Zhixing Luo, Wenjuan Wang, Xiaohu Xue, Bo Dong

**Affiliations:** 1 School of Engineering Sciences, University of Chinese Academy of Sciences, Beijing 100049, China; 2 Institute of Porous Flow and Fluid Mechanics, Chinese Academy of Sciences, Langfang 065007, China; 3 PetroChina Research Institute of Petroleum Exploration & Development, Beijing 100083, China; 4 Research Institute of Exploration and Development, PetroChina Xinjiang Oilfield Company, Karamay 834000, China; 5 China Petroleum Technology and Development Corporation, Beijing 100028, China

**Keywords:** CO_2_-soluble surfactant, mobility control, solubility, CO_2_-EOR

## Abstract

CO_2_ foam injection has been shown to be effective under reservoir conditions for enhanced oil recovery. However, its application requires a certain stability and surfactant absorbability on rock surface, and it is also associated with borehole corrosion in the presence of water. Adding surfactants to CO_2_ can enhance the interaction between CO_2_ and crude oil and control the CO_2_ mobility, thereby improving the performance of CO_2_ flooding. This paper presents a review of the research of CO_2_-soluble surfactants and their applications. Molecular dynamics simulation is introduced as a tool for analyzing the behavior of the surfactants in supercritical CO_2_ (scCO_2_). The applications of CO_2_-soluble surfactants, including CO_2_ thickening, reducing miscibility pressure, and generating supercritical CO_2_ foam, are discussed in detail. Moreover, some opportunities for the research and development of CO_2_-soluble surfactants are proposed.

## 1. Introduction

Supercritical CO_2_ is a carbon dioxide in a state above its critical point (304.13 K and 7.3773 MPa), where it has properties of both a gas and a liquid [1]. It exhibits high diffusivity, low viscosity, and low surface tension, making it an effective solvent for many organic compounds, such as oil, fat, and polymer. This environmentally friendly solvent is increasingly attractive to the petroleum industry as a replacement for traditional organic solvents [2,3]. It can be used in extraction [4], enhanced oil recovery (EOR) [5,6,7,8], CO_2_ sequestration [9], and cleaning processes [10,11].

CO_2_ flooding has been used for decades and is a proven technique to enhance the recovery of low- and ultralow-permeability reservoirs [12,13,14,15]. In the United States alone, CO_2_ flooding contributes 5.6% of the country’s total oil production, reaching 1371 × 10^4^ t, accounting for 93% of the world’s CO_2_-EOR oil production [16,17], and of the 136 CO_2_ flooding projects, low-permeability reservoir projects account for 63.67% [18]. Because of the low viscosity and low density of CO_2_, CO_2_ is prone to causing viscous fingering, premature breakthrough, unfavorable mobility ratios, gravity differentiation, low swept volumes, and other problems underground, seriously affecting the effectiveness of CO_2_-EOR. To improve the performance of CO_2_ flooding, it is critical to control the mobility of CO_2_.

The methods for controlling CO_2_ mobility mainly include water alternating gas (WAG) [19,20], gel profile control [21,22,23], direct thickeners (polymer and surfactant) [24], carbonated water injection (CWI) [25], micro and nanobubbles (MNB) [26], and surfactant-assisted or nanoparticle-assisted CO_2_ foam [27,28,29,30]. However, the contact of water with CO_2_ in these systems can lead to tubing corrosion and submersible pump corrosion, causing construction, sustainability, and cost issues [19]. The measurement of polymer and surfactant solubility is restricted by the uncertainty in cloud point determination [31], and highly soluble polymer and surfactants are often accompanied by high toxicity and high cost. WAG does not avoid gravity override and gel profile in not applicable to low permeability reservoirs. Table 1 provides the adverse effects of CO_2_ and mobility control methods. 

CO_2_ foam injection has been shown to be effective in a variety of reservoir conditions, including high temperature and high salinity [7,8]. However, its application for EOR requires a prior confirmation of several properties, including the stability of CO_2_ foam under reservoir conditions [32,33,34], the surfactant absorbability on rock surface [35,36,37], and the ability to maintain a stable foam in the presence of CO_2_ [38,39,40,41]. To address these challenges, researchers have developed various CO_2_-soluble surfactants with different molecular structures and properties. CO_2_-soluble surfactants are able to dissolve in scCO_2_ to produce a transparent, stable phase [42,43], which can increase the viscosity of scCO_2_ to a certain extent [44]. They are injected through cost-effective facilities, with no water produced [45]. Underground, surfactants and CO_2_ interact with crude oil to improve the sweep volume and efficiency. 

Eastoe et al. [46,47] reviewed the role of fluorocarbon surfactants in water-in-carbon dioxide (W/C) microemulsions. Enick et al. [29] reviewed the application of some surfactants in carbon dioxide thickening, but they were limited to fluorocarbon and silicone surfactants. Eastoe et al. [48] reviewed low-fluorine surfactants and non-fluorine surfactants in subsequent articles, but they did not mention the application of CO_2_-soluble surfactants in EOR. Therefore, we review the research of CO_2_-soluble surfactants through laboratory-scale and field-scale tests of EOR and propose the application prospects and challenges.

The adverse effects of CO_2_ and mobility control methods are listed in Table 1.

**Table 1:** Adverse effects of CO_2_ and mobility control methods.

Adverse Effect		Mobility Control Method	Shortcoming	Reference
Low viscosity	Viscous fingering	Water alternating gas (WAG)	It may cause tubing corrosion and scaling and not avoid gravity override.	[19,20]
	Premature breakthrough	Gel profile control	It is not applicable to low-permeability reservoirs.	[21,22,23,49,50]
	Unfavorable mobility ratio	Direct thickeners (polymer and surfactant)	High solubility is impossible under reservoir conditions.	[24]
Low density	Gravity override	Carbonated water injection (CWI) or micro and nanobubbles (MNBs)	It leads to low CO_2_ content and accelerated tubing corrosion.	[25,26]
	Low swept volume	Surfactant-assisted or nanoparticle-assisted CO_2_ foam	It induces adsorption during injection and tubing corrosion.	[27,28,29,30]

## 2. CO_2_-Soluble Surfactants

CO_2_ is a weak solvent with very low polarizability and a very low dielectric constant (ε = 1.1.0280 at 2.58 MPa and 25.05 °C [51]). Previous simulation studies showed that most surfactants are slightly or not at all soluble in scCO_2_ [52]. This finding is believed to be attributable to the stronger hydrophobic chain–to–hydrophobic chain interaction of the surfactant than the CO_2_ hydrophobic chain interaction. Hoefling et al. [53] enabled the solubility of a surfactant in CO_2_ by introducing functional groups with low polarity and low solubility parameters or those acting as Lewis bases on the CO_2_-hydrophobic tail chain [54].

A well-performed CO_2_-soluble surfactant is characterized by improved solubility and viscosity and enhanced interaction with crude oil to reduce oil–water interfacial tension or lower minimum miscibility pressure (MMP). Compared to conventional surfactants, CO_2_-soluble surfactants can reduce the mobility of the injected CO_2_ and the adsorption of surfactants on the rock surface significantly [55,56].

It is found that the addition of co-solvents (e.g., ethanol, 1-pentanol, F-pentanol, and tri-*n*-butylphosphate) increases the solubility of polar compounds in CO_2_ [57,58]. Liu et al. [59] studied the influence of alcohols on the phase behavior of a nonionic surfactant Ls-54/CO_2_ system. The results showed that the cloud point pressure (CPP) of the surfactant reduces significantly in the presence of 1-propanol, *n*-pentanol, and *n*-heptanol, and the reduction is more remarkable in the case of short-chain alcohols. Conversely, the addition of benzyl alcohol resulted in a CPP increase and solubility decrease of the surfactant. Chennamsetty et al. [60] investigated how alcohols affect the self-assembly of surfactants in scCO_2_ using lattice Monte Carlo simulations. They found that short-chain alcohols were concentrated in the surfactant layer of the aggregates, replacing the surfactant molecules, so they acted as co-surfactants, which directly affected the performance of the aggregates, while long-chain alcohols acted as co-solvents, changing the properties of the solvent.

Table 2 provides the main CO_2_-soluble surfactants discussed in this section.

### 2.1. Fluorocarbon Surfactants

Fluorocarbon surfactants were first discovered to be soluble in scCO_2_ [79]. CO_2_-soluble surfactants could be synthesized using perfluoroalkyl polyether carboxylate as tail groups instead of hydrocarbon carboxylate [80]. Harrison et al. [81] synthesized the first double-chain hybrid CO_2_-soluble surfactant composed of fluorocarbon (F) and hydrocarbon (H) chains. Cummings et al. [61] synthesized a series of semifluorinated F-H hybrid surfactants based on pentadecafluoro-5-dodecyl (F_7_H_4_) sulfate anion (M-F_7_H_4_, where M may be Li, Na, K, or Rb), which can generate more anisotropic micelles in scCO_2_ and exhibit a solubility up to 4.4 wt% in certain ranges of pressure and temperature.

Temtem et al. [62] investigated the interaction between fluorine atoms and CO_2_ molecules through nuclear magnetic resonance (NMR) and molecular simulation. They found that the higher solubility of perfluorinated molecules may be related to the fundamental differences in the nature of their interaction with CO_2_ compared to non-fluorinated molecules. In perfluorinated molecules, the carbon atom of CO_2_ acts as a Lewis acid, and the fluorine atoms act as Lewis bases in non-fluorinated molecules; the oxygen atom of CO_2_ acts as a Lewis base, and the proton of the hydrocarbon chain acts as a Lewis acid. Dardin et al. [82] used NMR spectroscopy to investigate the interactions between fluorocarbon solutes and scCO_2_ solvent. They found evidence of specific solute–solvent interactions, such as hydrogen bonding and van der Waals forces, which were different from the interactions observed in traditional solvents, such as water and organic solvents. They attributed this excess magnetic shielding to van der Waals interactions between the fluorinated sites in the solute and carbon dioxide.

Beckman [83] attributed the high solubility of fluorinated compounds in scCO_2_ to the following points: (1) the presence of fluorine creates molecules with weak self-interaction, making the miscibility with CO_2_ possible at low pressures; (2) electronegative fluorine may interact with electron-poor carbon of CO_2_, reducing the miscibility pressure; or (3) the presence of fluorine affects the acidity of adjacent protons, allowing the possibility of specific interactions between these protons and the oxygen atoms of CO_2_.

Mohamed et al. [63] synthesized a new hybrid surfactant CF_2_/AOT_4_ [sodium (4*H*,4*H*,5*H*,5*H*,5*H*-pentafluoropentyl-3,5,5-trimethyl-1-hexyl)-2-sulfosuccinate], which has one hydrocarbon chain and one fluorocarbon chain. This hybrid H-F chain structure strikes a fine balance of properties, thus minimizing the fluorine content and maintaining a sufficient level of CO_2_ solubility. Its solubility can reach 2.59 wt% at 34 MPa and 40 °C. Therefore, this structure and fluorination level can be used as a benchmark when designing low-fluorine surfactants.

Recently, the aggregation behavior and interfacial properties of a hybrid surfactant sodium 1-oxo-1-[4-(perfluoroalkyl)phenyl]alkane-2-sulfonates, FC_m_-HC_n_ (FC length m = 4, 6, HC length *n* = 2, 4, 5, 6, and 8) were examined in water–CO_2_ (W/C) mixture as functions of pressure and temperature. FC_6_-HC_4_ exhibited a higher solubility (up to 1.08 wt% at 35 MPa and 45 °C) than other surfactants [64,65].

Fluorocarbon surfactants are expensive and may cause environmental issues during subsurface applications [84,85,86], and those with perfluoroalkylpolyether tails also have toxicity concerns [87]. Therefore, they cannot be applied on a large scale.

### 2.2. Silicone Surfactants

Judicious side chain functionalization of oligomeric silicones has been shown to produce a material whose phase behavior in CO_2_ resembles that of fluorinated polyethers [88]. Hoefling [89] explored the relationship between the structure and solubility of silicone-based amphiphiles in CO_2_ via high-pressure phase-behavior experiments.

Alzobaidi et al. [90] used a comb polymer surfactant with a polydimethylsiloxane (PDMS) backbone and pendant linear alkyl chains to generate emulsions. Trisiloxane surfactants with very short and bulky CO_2_-philic headgroup forms showed extremely high solubility in CO_2_. For trisiloxane M(D’E_7_)M with seven EO repeat units, the solubility reached 1 wt% at 30 MPa and 25 °C [91].

Shi et al. [67] synthesized four siloxane polyether surfactants using allyl bromide, polyethylene glycol methyl ether, and heptamethyltrisiloxane (HMTS) as raw materials, which showed high solubility in CO_2_. In particular, HMTS demonstrated the highest solubility, around 2.4 mol%.

Silicone surfactants are also expensive and environmentally unfriendly. Efforts have been made to obtain low toxicity and less expensive CO_2_-soluble hydrocarbon-based surfactants, including hydrocarbon surfactants and oxygenated hydrocarbon surfactants [92,93,94,95].

### 2.3. Hydrocarbon Surfactants

Hydrocarbon surfactants developed for CO_2_ offer significant advantages over costly fluorocarbon and silicone surfactants. Hydrocarbon agents are the most promising CO_2_-soluble surfactants because of their environmentally friendly nature. The solubility of hydrocarbon surfactants in CO_2_ may be achieved by the addition of a polar co-solvent to CO_2_ to improve solvent polarity.

Hydrocarbon surfactants were first synthesized by Traian [96]. He used inexpensive propylene and CO_2_ to synthesize a series of poly(ether-carbonate) copolymers that readily dissolve in CO_2_ at low pressures.

Pitt et al. [97] pointed out that t-butyl chain tips promote the lowest aqueous surface tensions for hydrocarbon surfactants. Eastoe et al. [98] studied the solubility of octylphenol nonionic surfactants (TritonX-100, X-100 reduced, and X-45) chain end group structures (i.e., highly methylated tert-butyl units) in CO_2_ and aggregation effects. The results showed that the solubility of Triton surfactants depends on temperature and pressure, and methylation at the chain ends can promote the solubility in scCO_2_. Therefore, the t-butyl end can be considered a CO_2_-compatible group.

Sagisaka et al. [68] customized an isostearyl surfactant, sodium 2-(4,4-dimethylpentan-2-yl)-5,7,7-trimethyloctyl sulfate (SIS1), with a highly methyl-branched alkyl isostearyl group as a CO_2_-philic tail and observed the water–CO_2_ phase behavior at a fixed concentration of 0.08 mol%.

Shi et al. [99] measured the solubility pressure of three nonionic hydrocarbon surfactants (TX45 and TX100, Guerbet alkyl polyoxyethylene ether, and linear alkyl polyoxyethylene ether). The results showed that the surfactant with methylated tails (TX45 and TX100) had the highest solubility, and the surfactants with branched tails were less soluble than linear surfactants. TX45 exhibited a higher solubility (up to 0.188 wt% at 17 MPa and 70 °C) than those of other surfactants. Moreover, the addition of hexanol improved the solubility, which might be due to the fact that *n*-hexanol molecules can intercalate between the tails of the surfactant to hinder the interaction between molecules.

Liebum et al. [100] tested the solubility of three alkylamine surfactants in scCO_2_ and supercritical carbon dioxide–methane mixture (scCO_2_-scCH_4_) under high-pressure conditions at 40 °C and 60 °C. It was observed that highly methylated surfactant structures had the highest solubility in scCO_2_, up to 1 wt% at 40 °C, and the solubility decreased exponentially with the addition of methane to the system. Because of the strength of the surfactant–scCO_2_ intermolecular interaction, the scCO_2_ preferred shorter tail groups.

### 2.4. Oxygenated Hydrocarbon Surfactants

CO_2_ has a substantial quadrupole moment that operates over a much shorter distance than dipolar interactions [101,102]. It has been shown to have strong Lewis acid–Lewis base interactions with oxygen atoms of some ethers or carbonyls on solute molecules [103,104,105,106]. There are several oxygenated hydrocarbon groups that exhibit more favorable thermodynamic interactions with CO_2_ than branched alkanes. Accordingly, a series of acetylated sugars and cyclodextrins are highly soluble in scCO_2_ [92,107,108,109]. These surfactants, branched alkylphenol ethoxylates, branched alkyl ethoxylates, and a fatty acid–based surfactant, are also soluble in CO_2_ under the condition of good foaming performance [110].

The surfactant bis(2-ethylhexyl)sodium sulphosuccinate (AOT) is generally thought to be completely insoluble in scCO_2_ [81]. Ihara et al. [69] found that AOT can be completely dissolved in scCO_2_ containing 7.5% ethanol. Eastoe et al. [70,71,72] investigated the phase behaviors of AOT and its homologs and found that surfactants with trimethylpentyl or trimethylhexyl tails, sodium bis(2,4,4-trimethyl-1-pentyl) sulfosuccinate, and sodium bis(3,5,5-trimethyl-1-hexyl) sulfosuccinate are soluble in CO_2_.

Zhang et al. [111] used AOT as the CO_2_-soluble surfactant to stabilize CO_2_ foam in the presence of ethanol and demonstrated by core-flooding experiment that AOT dissolved in scCO_2_ can interact with formation water in situ to form scCO_2_ foam and thus control the mobility of CO_2_. Some scholars believe that the dissolution of surfactants in CO_2_ can lead to the formation of microemulsions and emulsions [71]. The low–molecular weight surfactant bis (3,5,5-trimethyl-l-hexyl) sodium sulfosuccinate (AOT-TMH) could stabilize diluted W/C emulsions with average diameters of 50 nm and 4 μm, respectively, for miniemulsions and macroemulsions. Also, AOT W/C reverse microemulsions could be formed in scCO_2_ by adding a small quantity of F-pentanol [112].

Gold et al. [113] found that the addition of a third chain to sulphosuccinate surfactants significantly increases the activity in CO_2_. Hollamby et al. [73] designed a triple hydrocarbon chain surfactant, sodium 1,4-bis(neopentyloxy)-3-(neopentyloxycarbonyl)-1,4-dioxobutane-2-sulfonate (TC14). TC14, with three CO_2_-philic chains, efficiently formed hydrated reverse micelles in scCO_2_, while conventional twin-tailed surfactants could not.

Ryoo et al. [74] investigated the formation of W/C microemulsions by nonionic methylated branched hydrocarbon surfactants, poly(ethylene glycol) 2,6,8-trimethyl-4-nonyl ethers. They found that methylation and branching increased the solubility of surfactants in CO_2_ by weakening the interactions between the tails.

Fan et al. [92] synthesized a series of oxygenated hydrocarbon-tailed ionic surfactants composed of acetylated sugar, poly-p-phenylene oxide, or oligo(vinyl acetate) and evaluated their solubility in scCO_2_. They found that the oligo(vinyl acetate)-functionalized surfactants were highly soluble in scCO_2_, with single-tailed surfactants having the solubility of 7 wt% at 25 °C and 48 MPa and twin-tailed surfactants having the solubility of 3 wt%.

Liu et al. [93] studied the solubility of the acetylene glycol–based nonionic surfactant Dynol-604 (a non-fluorine and non-silicone surfactant) in scCO_2_. They observed that Dynol-604 had a solubility that increased with rising pressure and decreased with elevating temperature, which reached 5 wt% at 26 MPa and 60 °C. They also investigated non-fluorine and non-silicone nonionic surfactants Ls-36 and Ls-45 containing propylene oxide (PO) and ethylene oxide (EO) groups and found that both Ls-36 and Ls-45 were highly soluble in scCO_2_ [94] and that an increase in the number of EO groups reduced the solubility in CO_2_ [98]_._

Burrows et al. [75] evaluated the CO_2_ solubility of three nonionic surfactants (branched tridecyl ethoxylate Indorama SURFONIC TDA-9, branched nonylphenol ethoxylate Indorama SURFONIC N-100, and linear dodecyl ethoxylate Indorama SURFONIC L12-6). The results showed that each surfactant could dissolve in CO_2_ up to 1 wt% at pressures and temperatures commensurate with CO_2_ EOR.

Zhang et al. [57] investigated the dissolution of surfactants in scCO_2_ in the presence of co-solvents, nonionic surfactants (N-NP-10c, branched alkylphenol ethoxylates and APG-1214, Alkyl polyglucoside), and anionic surfactants (N-NP-15c-H, sulfonated alkylphenol ethoxylates). The results showed that increasing the pressure and adding co-solvents could effectively improve surfactants’ dissolution in CO_2_, and the dissolution of surfactants and co-solvents in CO_2_ solutions could greatly increase the viscosity of the mixture.

Chen et al. [76,77,78] proved that the thermally stable amine ethoxylate C_12–14_N(EO)_2_ is a well-performed candidate foam agent that can dissolve in CO_2_ even in high salinity at high temperature. They indicated that C_12–14_N(EO)_2_ is switchable from a nonionic to a cationic state by lowering pH and soluble in brine when it is cationic and in scCO_2_ when it is nonionic. They also reported that 0.2 wt% C_12–14_N(EO)_2_ and C_12–14_N(EO)_5_ can still be dissolved in CO_2_ at a pressure ˂23 MPa and a temperature up to 120 °C. Zhang et al. [114] used the tallow ethoxylated amine surfactant, C_16–18_N(EO)_5_, to generate and stabilize CO_2_ foam at high pressure and high temperature. They found that C_16–18_N(EO)_5_ is soluble in scCO_2_ up to 0.5 wt%, and it is effective in improving foam stability when it is added during the CO_2_ phase.

The dissolution of oxygenated hydrocarbon surfactants requires a large amount of co-solvent, which adds cost. In addition, co-solvent reduces the viscosity of the system, which is not conducive to mobility control. It is necessary to seek an optimal addition of co-solvent to minimize cost while meeting the required solubility.

## 3. Molecular Dynamics Simulation

Molecular dynamics (MD) simulations provide us with an atomic-level insight into the surfactant solubility in scCO_2_. Salaniwal et al. [115] reported the first molecular simulations of the self-assembly of di-chain surfactants in scCO_2_ into stable, spherical aggregates. Rocha [116] investigated the structural properties of the W/C binary fluid−fluid interface by means of MD simulation.

Lísal et al. [117] modified Larson’s lattice model and used it to study micellar behavior in a supercritical solvent–surfactant system via large-scale Monte Carlo simulations. Li et al. [118] performed simulations on model homopolymer/solvent systems with varying interaction strengths and explored the influence of surfactant structure (head and tail lengths) on phase transition. Ren et al. [119] studied the microstructure of CO_2_ microemulsions via MD simulations, revealing the origin of the synergistic effect between hydrotropes and surfactants. Zhu et al. [120] reported the dynamic process and interface equilibrium structure in self-assembled aggregates based on MD simulations.

Zhang et al. [121] used MD simulations to clarify the AOT–AOT and AOT–solvent interactions and their effects on interfacial properties, such as interfacial tension (IFT) and interfacial thickness at the molecular level, which indicated that ethanol enhances the foam stability and regeneration capacity of CO_2_-soluble surfactant. Nan et al. [122,123] investigated how alcohols with different tail lengths (C_2_OH-C_16_OH) and concentrations affect the water–AOT–scCO_2_ interface system through MD simulations. Kobayashi et al. [124] discussed the mutual solubility of heavy *n*-alkanes (typically, *n*-decane, *n*-hexadecane, *n*-eicosane) and their structural isomers in CO_2_-rich and hydrocarbon-rich phases using continuous fractional component Gibbs integrated Monte Carlo simulations and suggested that the improvement of solubility is due to a higher coordination number of CO_2_ for methyl (CH_3_) rather than for methylene (CH_2_) groups.

MD simulations allow the in-depth analysis of the interactions between CO_2_-soluble surfactants and gases in order to understand the behaviors of surfactants in the gas phase and thus optimize their performance.

## 4. Applications

CO_2_-soluble surfactants can be applied for CO_2_ thickening, reducing minimum miscibility pressure (MMP), and generating scCO_2_ foam. This section focuses on the critical roles of surfactants in controlling the CO_2_ properties in the reservoir, thereby enhancing oil recovery. Table 3 provides the summary of studies on the applications of CO_2_-soluble surfactants for EOR.

### 4.1. CO_2_ Thickening

Self-assembly of surfactants can form linear or rod-like micelles, which, at a certain concentration, will intertwine to form a network structure, thereby increasing the viscosity of scCO_2_ [138].

Trickett et al. [125] synthesized a fluorinated surfactant Ni(di-HCF4) by ion exchange and measured its viscosity using a falling ball viscometer (10 wt% Ni(di-HCF4) at 25 °C and 35 MPa). The results showed that the viscosity of the system increased by 90% compared to pure CO_2_, to 0.22 MPa·s, suggesting that surfactant self-assembly can control the viscosity of CO_2_.

Zhao et al. [126] investigated the solubility and thickening properties of three polysiloxanes modified with different functional groups in scCO_2_. The results showed that vinyl polysiloxane had the highest solubility and best thickening capacity, and the higher the kinematic viscosity and concentration of vinyl polysiloxane, the better the thickening capacity. It was also indicated that, at 40 °C and 39.24 MPa, adding 8 wt% (1000 centistokes) vinyl polysiloxane enabled the viscosity of the scCO_2_ system to reach 12.57 MPa·s.

Other researchers discussed factors such as molecular weight, concentration, shear rate, temperature, and pressure that affect CO_2_ thickening. Table 4 [24,139] presents a summarization of these factors.

### 4.2. Reducing Miscibility Pressure

CO_2_-soluble surfactants improve CO_2_-oil miscibility mainly by mitigating the IFT of the oil–gas system, increasing the volume expansion of oil, and reducing the viscosity of oil.

Dong et al. [127] prepared a scCO_2_ microemulsion system by adding CO_2_-soluble surfactant AOT and co-solvent ethanol to CO_2_ and found that the MMP of the CO_2_-oil system was reduced from 24.55 to 22.02 MPa.

Wang [128] synthesized different fatty alcohol polyethers and demonstrated that the fatty alcohol polyoxypropylene ether performed better in reducing the MMP during CO_2_ flooding than the polyoxyethylene ether. In other words, PO is more soluble in CO_2_ than EO, and it is more CO_2_-philic.

Guo et al. [129] synthesized an oil-soluble surfactant CAE and evaluated its effects on the IFT and MMP of the CO_2_–oil system through experiments. The results showed that CAE is soluble in scCO_2_ but insoluble in water, reducing the MMP by 22.34% at a concentration of 0.2 wt%.

Zhang et al. [130] investigated the synergy between ethanol and surfactants in lowering the IFT and MMP of the CO_2_–oil system. They indicated that, under the optimal conditions, that is, 7 wt% ethanol + 0.3 wt% TXIB(2,2,4-trimethyl-1,3,-pentaerediol diisobutyl ester), the MMP was reduced by as much as 30.2%.

Lv et al. [131,132] treated different nonionic polyether surfactants for their solubility in CO_2_ and their potential to improve oil–gas miscibility. The results showed that polyoxypropylene alkyl ethers, especially those with low molecular weights and high PO groups, significantly improved the oil–gas miscibility. Under the conditions of 50 °C, adding 3 wt% C_4_PO_3_ could reduce MMP from 17.75 to 13.6 MPa.

Kuang et al. [140] dealt with five surfactants in terms of their solubility in CO_2_ and their improvements in CO_2_-oil miscibility. The results indicated that surfactants could reduce the viscosity of oil to a certain extent and lower the IFT between CO_2_ and oil, thereby enhancing the miscibility of the two phases. This effect would be further enhanced after adding low-carbon alcohols. Under the conditions of 50 °C and 30 MPa, adding 0.5 wt% SPO5 and 0.25 wt% *n*-pentanol to CO_2_ increased the recovery by 2.29% compared with CO2 alone, ultimately reaching 93.47%.

Li et al. [141] selected two nonionic alkoxylated surfactants (ethylene glycol butyl ether and Span 80) to analyze the MMP reduction in CO_2_–oil systems. The results showed that the addition of surfactants could accelerate the miscibility process and change the rock surface from oil-wet to water-wet, thereby promoting the oil flow in the reservoir and ultimately enhancing oil recovery.

### 4.3. Supercritical CO_2_ Foam

Conventional foaming surfactants only traverse through the reservoirs in the aqueous phase [41]. Surfactants required for foam generation and stabilization can be injected by dissolving in an aqueous solution [142]. CO_2_-soluble surfactants are soluble in scCO_2_, and their injection does not involve water, which can mitigate the risk of wellbore corrosion. The surfactants dissolved in CO_2_ can interact in situ with formation water in the presence of CO_2_ to form CO_2_ foam, which helps reduce the mobility of CO_2_ and improve the sweep efficiency, thereby enhancing oil recovery.

Le et al. [133] proposed for the first time that foam can be generated in situ via the injection of surfactant in the CO_2_ phase. SACROC [56] carried out a pilot test on a CO_2_ foam system with CO_2_-soluble surfactant, revealing an effective control on the CO_2_ mobility and reduction of the oil–gas ratio.

The high solubility of a surfactant in CO_2_ and a favorable W/C partition coefficient are beneficial for the transport of the surfactant along CO_2_-flow pathways in the reservoirs to minimize the possibility of viscous fingering and gravity override. Ren et al. [16,134] studied the effect of surfactant partitioning between scCO_2_ and water on surfactant transport and foam propagation during two-phase flow. They found that, for all CO_2_-soluble surfactants studied, the core-scale CO_2_ displacement rates increased with decreasing surfactant partition coefficients.

Bi et al. [135] evaluated the solubility of 31 oilfield or industrial surfactants and their modified products in scCO_2_ using a high-pressure and high-temperature visualized foam device. The results showed that the surfactant N-P-12 had good CO_2_ foam stability at high temperature (120 °C) and the addition of alcohol as co-solvent could significantly increase the solubility of the surfactants in the CO_2_.

The Baker Hughes researchers [143] suggested the use of CO_2_ foams for gas lift operations in conjunction with a cross-linked siloxane polymer (Dow Corning 1250 (poly(trimethylhydrosilylsiloxane), MW 5770, MN 3160) as the recommended surfactant.

Ramadhan [136] investigated the foaming behavior of a CO_2_-soluble, cationic, amine-based surfactant, N.N.N’-trimethyl-N’-tallow-1,3-diaminopropane (DTTM), which forms wormlike micelles at elevated salinity. They believed that the viscous fingering of surfactant-carrying CO_2_ causes a delay in foam generation and propagation.

Haeri et al. [137] measured the CO_2_ solubility of two nonionic, water-soluble, branched alkyl tail surfactants (Indorama SURFONIC^®^N-100, an ethoxylated nonylphenyl alcohol with 10 ethylene oxide groups, and Indorama SURFONIC^®^ TDA-9, an ethoxylated branched tridecyl alcohol with nine ethylene oxide groups) through core-flooding experiments at 25–75 °C. The results indicated that the surfactants had a solubility of roughly 1 wt%.

Burrows et al. [75] evaluated the CO_2_ solubility of three nonionic surfactants (branched tridecyl ethoxylate Indorama SURFONIC TDA-9, branched nonylphenol ethoxylate Indorama SURFONIC N-100, and linear dodecyl ethoxylate Indorama SURFONIC L12-6). They found that each surfactant dissolved in CO_2_ up to 1 wt% at pressures and temperatures comparable to CO_2_ EOR and CO_2_-dissolved surfactants did not significantly affect CO_2_–oil IFT or generate CO_2_ foams. They also reported that SURFONIC TDA-9 achieved the highest oil recovery of 75%, compared to 71% by pure CO_2_.

The partitioning of surfactant into the CO_2_ phase results in faster foam propagation and stronger foam [144]. After the foam collapses in the transport process, CO_2_-containing surfactants keep contact with formation water during the upward movement, which allows for the regeneration of foam and the resistance to foam collapse. This makes it possible to achieve effective control of CO_2_ mobility.

## 5. Outlook

The utilization of CO_2_-soluble surfactants for EOR presents both challenges and opportunities. Despite the promising results observed in experimental studies and field tests, CO_2_-soluble surfactants cannot be applied widely unless some limitations are addressed. Fluorinated and siloxane surfactants have notable solubility in CO_2_, but they come with high cost and toxicity. Hydrocarbon and oxygenated hydrocarbon surfactants exhibit low solubility in scCO_2_, so they can work only with the support of a large amount of co-solvent. These challenges hinder the large-scale field application of CO_2_-soluble surfactants.

Nonetheless, new surfactant molecules can be tailored for diverse applications. Such efforts should be made to enhance the solubility in CO_2_, minimize the necessity of co-solvent, and improve the performance in IFT and foam stability. Rigorous investigations into the molecular interactions between CO_2_ and surfactants are crucial for engineering formulations that effectively lower IFT. The systematic screening of surfactant structures and functional groups can help define the configurations yielding the highest IFT reduction under reservoir conditions. Foam stability can be optimized by adjusting the surfactant concentration, formulation composition, and inclusion of additives such as CO_2_-soluble polymers.

Additionally, there is a growing interest in the development of biomass-based CO_2_-soluble surfactants or renewable surfactants, which are cost-effective and environmentally friendly.

## 6. Conclusions

This paper reviews the research of CO_2_-soluble surfactants and their applications for EOR and proposes the relevant challenges and opportunities.

The addition of CO_2_-soluble surfactants has been shown to be promising in mitigating the adverse effects of CO_2_ injection to enhance oil recovery. By modifying the structures and concentrations of CO_2_-soluble surfactants, it is possible to achieve CO_2_ thickening, reduce the miscibility pressure, and generate scCO_2_ foam. CO_2_-soluble surfactants can help enhance oil recovery by improving viscosity, reducing IFT, and increasing sweep efficiency in the reservoir. Molecular dynamics simulations provide researchers with theoretical and predictive insights into the screening and design of CO_2_-soluble surfactants with lower costs of trial and error for the purpose of efficient application.

While experimental studies demonstrated significant improvements in oil recovery, some CO_2_-soluble surfactants are challenging for application because of their high cost and toxicity, as well as solubility-related issues and co-solvent usage. Future research should focus on the development of new surfactant molecules, optimization of formulations, and determination of environmentally friendly alternatives.

## Figures and Tables

**Table 2:** Structures of CO_2_-soluble surfactants discussed in this section.

Structure	Conditions	Solubility	Co-Solvent	Reference
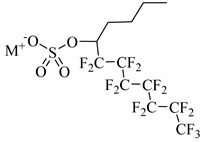 M-F7H4	60 MPa 45 °C	4.4 wt%	-	Cummings [61]
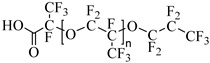 Krytox 157 FSL Mw 2500	22 MPa 60.85 °C	1.72 wt%	-	Temtem [62]
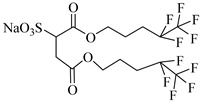 di-CF2	22.4 MPa 40 °C	2.77 wt%	-	Mohamed [63]
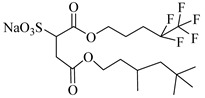 Hybrid CF2/AOT4	34 MPa 40 °C	2.59 wt%	-	Mohamed [63]
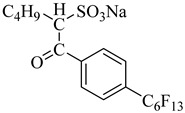 FC_6_-FC_4_	35 MPa 45 °C	1.08 wt%	-	Sagisaka [64,65]
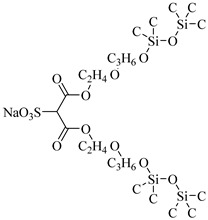 Sulfonated siloxane-functional sulfonate surfactants	31.7 MPa 65 °C	1.00 wt%	-	Fink [66]
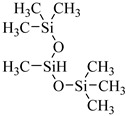 HMTS	9.25 MPa 50 °C	0.11 wt%	-	Shi [67]
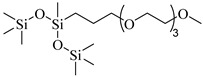 (PEG)_3_-TS	10.09 MPa 50 °C	0.003 wt%	-	Shi [67]
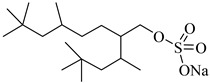 SIS1	35–75 °C 10–40 MPa	0.007 wt%	-	Sagisaka [68]
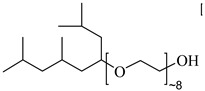 TMN-6	35–75 °C 10–40 MPa	0.004 wt%	-	Sagisaka [68]
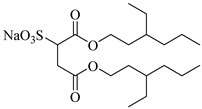 AOT	30 MPa 60 °C	Dissolved	Ethanol	Ihara [69]
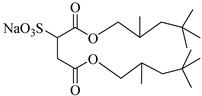 sodium bis(2,4,4-trimethyl-1-pentyl)sulfosuccinate	25 MPa 33 °C	Dissolved	-	Eastoe [51,52,53]
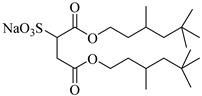 sodium bis(3,5,5-trimethyl-1-hexyl) sulfosuccinate	25 MPa 33 °C	Dissolved	-	Eastoe [70,71,72]
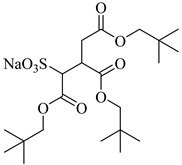 TC14	3 MPa 25 °C	Dissolved	-	Hollamby [73]
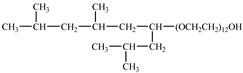 Tergitol TMN ethoxylated nonyl ether	2.75 MPa 25 °C	1.0 wt%	-	Ryoo [74]
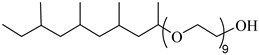 SURFONIC TDA-9	29 MPa 77 °C	1.0 wt%	-	Burrows [75]
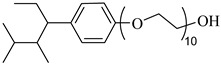 SURFONIC N-100	38 MPa 77 °C	1.0 wt%	-	Burrows [75]
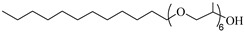 SURFONIC L12-6	31 MPa 77 °C	1.0 wt%	-	Burrows [75]
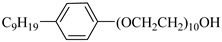 N-NP-10c	18.4 MPa 40 °C	1.76 wt%	Ethanol	Zhang [57]
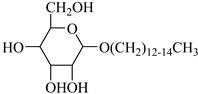 APG-1214	17.4 MPa 40 °C	1.66 wt%	Ethanol and ethylene glycol (3:2)	Zhang [57]
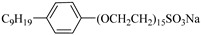 N-NP-15c-H	17 MPa 40 °C	0.57 wt%	Ethanol and ethylene glycol (4:1)	Zhang [57]
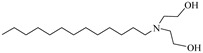 C_12–14_N(EO)_2_	22.76 MPa 120 °C	0.2 wt%	-	Chen [76,77,78]
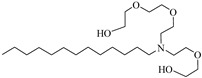 C_12–14_N(EO)_5_	17.93 MPa 120 °C	0.2 wt%	-	Chen [76,77,78]

**Table 3:** Summary of studies on the applications of CO_2_-soluble surfactants for EOR.

Reservoir Type	Conditions	System Composition	Function	Ultimate Oil Recovery	Reference
\*	25 °C, 35 MPa	Ni(di-HCF4) (10 wt%) in CO_2_	Thicken the system to 0.22 MPa·s	\	Trickett [125]
\	40 °C, 39.24 MPa	Vinyl polysiloxane (8 wt%) in CO_2_	Thicken the system to 12.57 MPa·s	\	Zhao [126]
Daqing crude oil	45 °C, 22,7 MPa	AOT (0.005 wt%–0.015 wt%), ethanol (13.76 wt%), water (0.41 wt%–1.61 wt%) in CO_2_	Reduce the MMP from 23.8 to 22.7 MPa	About 80%	Dong [127]
Shengli crude oil	60 °C, 13.22 MPa	C_12_PO_6_ (0.6 wt%), ethanol (0.7 wt%) in CO_2_	Reduce the MMP from 17.79 to 13.22 MPa	\	Wang [128]
\	85 °C, 21.2 MPa	CAE (0.2 wt%) in CO_2_	Reduce the MMP from 27.3 to 21.2 MPa	92.06%	Guo [129]
Shengli crude oil	60 °C, 11.41 MPa	TXIB (0.3 wt%%), ethanol (7 wt%) in CO_2_	Reduce the MMP from 16.79 to 11.41 MPa	\	Zhang [130]
\	50 °C, 13.6 MPa	C_4_PsO_3_ (3 wt%) in CO_2_	Reduce the MMP from 17.75 to 13.6 MPa	\	Lv [131,132]
QS8 oilfield	76 °C, 30 MPa	SPO5 (0.5 wt%), *n*-pentanol (0.25 wt%) in CO_2_	Reduce the IFT of the CO_2_-oil system	93.47%	Kuang [116]
Midland Farm (West Texas) crude oil	26.67 °C, 10.34 MPa	Surfactant (0.5 wt%) in CO_2_	Generate foam, as indicated by the immediate increase of pressure drop at the start of CO_2_ injection	60%	Le [133]
SACROC Field	60 °C, 24.13 MPa	ELEVATE™ CO_2_ EOR Conformance Solutions	Generate foam	Oil production increased by 30% in one month	Sanders [56]
Silurian-dolomite cores	35 °C, 10.34 MPa	CO_2_-soluble surfactants (S, 4 S, and 15 S) (0.15 wt%) in CO_2_	Reduce the delay of foam propagation	\	Ren [16,134]
\	120 °C, 20 MPa	N-P-12 (2.99 wt%), ethylene glycol (16.97 wt%) in CO_2_	Increase and then decrease the resistance	92.5%	Bi [135]
\	40 °C, 11.72 MPa	DTTM (0.5 wt%) in CO_2_	Cause delay in foam generation and propagation by viscous fingering	\	Ramadhan [136]
Eagle Ford outcrop rock Eagle Ford crude oil	80 °C, 27.6 MPa	SURFONIC^®^N-100 (0.1 wt%) in CO_2_	Alter the wettability of unconventional rock	75%	Haeri [137]

* “\” indicates that this reference does not provide this part of the parameters or results.

**Table 4:** General effects of factors on the solubility and viscosity of CO_2_.

Factor	General Effects on CO_2_ of Increasing the Factor	
	Solubility	Viscosity
Molecular weight	Decrease	Increase
Concentration	Increase and then decrease	Increase
Shear rate	Increase	Decrease
Temperature	Decrease	Decrease
Pressure	Increase	Increase

## Data Availability

Not applicable.
